# Electroacupuncture alleviates diabetic neuropathic pain in rats by suppressing P2X3 receptor expression in dorsal root ganglia

**DOI:** 10.1007/s11302-020-09728-9

**Published:** 2020-10-03

**Authors:** Xueyu Fei, Xiaofen He, Zhaoxia Tai, Hanzhi Wang, Siying Qu, Luhang Chen, Qunqi Hu, Jianqiao Fang, Yongliang Jiang

**Affiliations:** 1 Key Laboratory of Acupuncture and Neurology of Zhejiang Province, Department of Neurobiology and Acupuncture Research, The Third Clinical Medical College, Zhejiang Chinese Medical University, Hangzhou, 310053 China; 2 Zhejiang Chinese Medical University, Hangzhou, 310053 China

**Keywords:** Diabetic neuropathic pain, Streptozotocin, Electroacupuncture, P2X3 receptor, DRG

## Abstract

Diabetic neuropathic pain (DNP) is a troublesome diabetes complication all over the world. P2X3 receptor (P2X3R), a purinergic receptor from dorsal root ganglion (DRG), has important roles in neuropathic pain pathology and nociceptive sensations. Here, we investigated the involvement of DRG P2X3R and the effect of 2 Hz electroacupuncture (EA) on DNP. We monitored the rats’ body weight, fasting blood glucose level, paw withdrawal thresholds, and paw withdrawal latency, and evaluated P2X3R expression in DRG. We found that P2X3R expression is upregulated on DNP, while 2 Hz EA is analgesic against DNP and suppresses P2X3R expression in DRG. To evaluate P2X3R involvement in pain modulation, we then treated the animals with A317491, a P2X3R specific antagonist, or α β-me ATP, a P2X3R agonist. We found that A317491 alleviates hyperalgesia, while α β-me ATP blocks EA’s analgesic effects. Our findings indicated that 2 Hz EA alleviates DNP, possibly by suppressing P2X3R upregulation in DRG.

## Introduction

There are about 415 million diabetes cases worldwide, and the number is projected to rise to about 700 million by 2045 [1]. The global burden of diabetes mellitus (DM) severely affects quality of life and imposes tremendous healthcare burdens. Diabetic neuropathic pain (DNP) is a common, troublesome diabetes complication that affects 11–21% of diabetics [2] and is associated with numbness, spontaneous pain, hyperalgesia, and allodynia [3, 4]. DNP management is usually by analgesics, including pregabalin, duloxetine, and opioids [5, 6], but these are not always effective. Thus, a better understanding of the mechanisms underlying DNP is needed.

P2X3 receptor (P2X3R) belongs to the purinergic receptor (P2X) family and is highly expressed in the small- and medium-diameter sensory neurons of dorsal root ganglions (DRGs) [7–9]. Adenosine 5′-triphosphate (ATP) releasing from damaged cells in peripheral tissues mediates the activation of P2X receptors on cell surface, which contributes to inflammation [10, 11]. P2X receptor antagonists are known to inhibit mechanical allodynia in rats and mice [12, 13]. Neuropathic pain is often caused by somatosensory nervous system damage, causing the release of vast amounts of ATP. The expression of P2X3R is markedly high in DRGs [14, 15], and its inhibition relieves mechanical hyperalgesia in a neuropathic pain rat model [16]. Additionally, it is reported that P2X3R significantly enhances ATP-activated gated cation channel currents in sensory neurons [17, 18]. Highlight the P2X3R as a novel target for pain treatment.

Streptozotocin (STZ) is an antibiotic that can selectively destroy pancreatic islet β-cell [19]. STZ is widely used to generate diabetic animal models that recapitulate the features of human diabetes, including insulin deficiency, weight loss, hyperglycemia, polydipsia, polyuria, and polyphagia [20, 21]. Relative to diabetes animal models created by other means, including genetic induction of spontaneous diabetes [22, 23], or high-fat diet (HFD) combined with low-dose STZ [24, 25], STZ-induced models are more widely used to study DNP mechanisms and potential therapies, owing to its convenience and lower model generation costs.

Electroacupuncture (EA) is widely used to treat chronic pain, including diabetes-associated pain [26–28]. Mounting evidence in animal models indicates involvement of P2X3 receptor in neuropathic pain [29–33]. However, few DNP models are available. Previously, we have shown that EA suppresses P2X3R overexpression in DRGs and reduces DNP in type 2 DM rats [34]. Here, we created type 1 DM rats by a single large dose injection of STZ (65 mg/kg) and characterized P2X3R expression at various DNP stages. We then investigated if EA alleviates DNP by suppressing P2X3R expression in DRG neurons.

## Materials and methods

### Animals

Male Sprague-Dawley (SD) rats, weighing 160–200 g, were purchased from Shanghai Laboratory Animal Center of Chinese Academy of Sciences, (SCXK (hu) 2018-0006), and 5 rats per cage were housed in controlled 12-h light/dark cycles, at 24 ± 2 °C and 40–60% relative humidity with ad libitum access to water and food. Approval for animal experiments was provided by the animal welfare committee of Zhejiang Chinese Medical University (IACUC-20180723-08). The suffering of animals and their numbers was minimized by all efforts.

### Establishment of the type 1 DNP rat model

To induce DNP, animals were fasted overnight and then i.p. injected with STZ (Sigma-Aldrich, Cat. No. S0130-1G) in 0.1 M citrate buffer, pH 4.5, at 65 mg/kg of body weight [35, 36]. Control animals were injected with same volume of vehicle. After 7 days, rats with > 13.9 mmol/L of fasting blood glucose (FBG) were selected as type 1 diabetic rats [37, 38].

### Experimental design

**Fig. 1:**
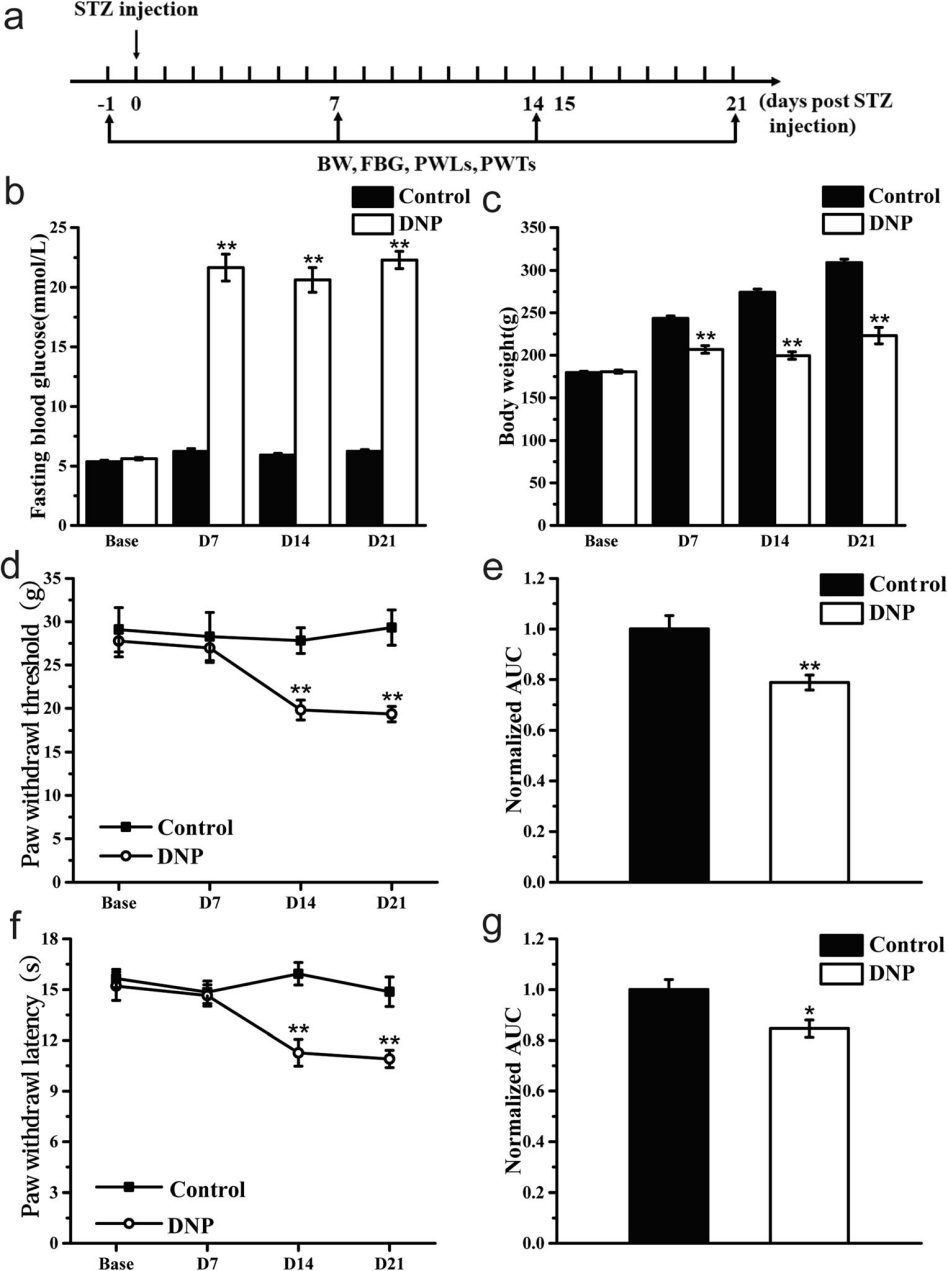
Generation of the DNP rat model. **a** Schematic of the DNP rat model creation process; **b** time course effect of STZ injection on FBG; **c** time course effect of STZ injection on body weight; **d** time course effect of STZ injection on PWT; **e** normalized area under the curve (AUC) analysis of **d**; **f** time course effect of STZ injection on PWL; **g** normalized area under the curve (AUC) analysis of **f**. AUCs were all normalized to the corresponding Control group. *n* = 6. *P < 0.05, **P < 0.01 vs. Control group

First, STZ effects were evaluated at 7, 14, and 21 days after STZ treatment by monitoring DNP and P2X3R expression on DRGs. Rats were randomly divided into a Control group (*n* = 6, sacrificed 21 days after STZ injection and tissue harvested) and a DNP group (*n* = 12, 3 rats were sacrificed 7 days after STZ injection for tissues, 3 rats were sacrificed 14 days after STZ injection for tissues, 6 rats were sacrificed 21 days after STZ injection for tissues). Paw withdrawal thresholds (PWT) and paw withdrawal latency (PWL) were examined according to the schedule (Fig. 1a).

Next, the involvement of P2X3R in DNP was evaluated by treating the animals with A317491, a P2X3 antagonist. Rats were randomly divided into a Control + NS group (*n* = 12), a DNP + NS group (*n* = 12), and a DNP + A317491 group (*n* = 12). The antagonist was administered on day 15 after STZ injection, with PWTs and PWLs being recorded according to the schedule (Fig. 4a).

The effects of 2 Hz EA on DNP and P2X3R expression in DRGs were assessed. Rats were randomly divided into a control group (*n* = 12), a DNP group (*n* = 12), and a DNP + EA group (*n* = 12). They were then treated with EA for 7 days and pain behavioral tests conducted according to the schedule (Fig. 5a). After 7 days post-EA treatment, rats were sacrificed for tissues.

Next, the role of DRG P2X3R on 2 Hz EA analgesic effects in DNP was examined. Rats were randomly divided into a Control + α β-me ATP group (*n* = 8), a DNP + α β-me ATP group (*n* = 8), and a DNP + EA + α β-me ATP group (*n* = 8). They were then treated with α β-me ATP, a P2X3 agonist for 7 days after EA treatment, and examined for whether P2X3 receptor reverses the 2 Hz EA analgesic effects (Fig. 7a).

### Behavioral analysis

#### Assessment of thermal pain sensitivity

Behavioral analysis of thermal pain sensitivity was examined by paw withdrawal latency (PWL) assay 1 day prior to STZ injection and then 7, 14, and 21 days after injection, until sacrifice. For PWL examination, rats were acclimated for 10 min to individual Plexiglas cubicles (17′ 22′ 14 cm) on the surface of a 2-mM-thick glass plate. Heat stimulus was then applied to the plantar surface of the left hind paw using the plantar test (37370, Ugo Basile, Italy). The cut-off time was set at 20 s and the radiant heat at 40 to avoid tissue damage. Each hind paw was independently tested 5 times at 5-min intervals. Baseline thermal withdrawal latency was recorded before STZ injection. Mean PWL was calculated by averaging the latencies of tests after removing the maximum and minimum values taken for data analysis.

#### Assessment of mechanical pain sensitivity

Mechanical pain sensitivity was evaluated using the paw withdrawal threshold (PWT) assay at the time of PWL analysis. We employed the dynamic plantar esthesiometer (37450, Ugo Basile, Italy), which is an automated tool that works on the principle of von Frey filament principle and assesses the PWT. The tool measured the sensitivity to touch stimuli. Rats were allowed to familiarize with the Plexiglas cubicles (17′ 22′ 14 cm) on a wire mesh platform for 10 min after which a probe that generates stimulations was placed under the hind paw. We then applied a vertical force (incrementally from 0 to 50 g in 20 s) to the paw and recorded the force that generated a limb withdrawal. The tolerance threshold was presented as the mean of 3 readings. The same protocol was followed to perform postoperative tests and measure the threshold to mechanical stimulus.

#### Persistent spontaneous nociception assessment

Evaluation of spontaneous nociception began immediately after α β-me ATP injection. Briefly, animals were habituated in Plexiglas cubicles situated over an HD camera (Sony, HDR-CX405) for 10 min and persistent spontaneous nociception (PSN) was estimated by counting the number of paw flinches. Paw flinches were counted at 2-min intervals. Pain-like behavior, including shaking, biting, and licking of the injected paw, was counted as paw flinches in 2-min bins across the entire 10-min test.

#### Immunofluorescence

Rats were deeply anesthetized by i.p. injection of sodium pentobarbital (80 mg/kg) and perfused with saline followed by 4% PFA in PBS1X (pH 7.4). The bilateral L4–6 DRGs were then removed and post-fixed in 4% PFA for 4 h before being dehydrated in 15% and 30% sucrose solution for 48 h (until sinking at 4 °C was observed). Tissues were sectioned at 10 μm using a frozen microtome and mounted onto glass slides. Sections were rinsed with TBST (pH 7.4) and blocked in 10% normal donkey serum in TBST (0.1% Tween-20) for 60 min at 37 °C. They were then incubated at 4 °C overnight with rabbit anti-P2X3 (Alomone, APR-016) alone at 1:400 or mixed with mouse anti-NeuN (Abcam, ab104224) at 1:600, mouse anti-GFAP (Sigma, G3893) at 1:400, or mouse anti-CD11 (Abcam, ab1211) at 1:500. After six 10-min washes with TBST (pH 7.4), the sections were incubated with Alexa Fluor 488 donkey anti-rabbit IgG (Jackson, 711-545-152) or Alexa Fluor 647 donkey anti-mouse IgG (Jackson, 715-605-150) at 1:800 for 1 h at 37 °C. The sections were then imaged on an A1R confocal microscope (Nikon) using a × 10 objective. Image analysis was done using ImageJ. Positive cells were counted in 3–5 random sections from each rat. Three rats were analyzed for each group.

#### Drug administration

A317491 (Sigma-Aldrich, A2979-5MG) and α β-me ATP (Sigma-Aldrich, M6517-5MG) were freshly dissolved in sterile 0.9% saline and diluted to required concentrations before each experiment. A317491 (50 ul, 100 nmol) and α β-me ATP (50 ul, 100 nmol) were administered via intraplantar injection into the left hind paw. Other groups received the same volumes of sterile saline.

#### Electroacupuncture

For electroacupuncture, rats were restrained without anesthesia. Acupuncture needles (0.25 mm*13 mm, Huatuo, Suzhou Medical Appliance Manufactory, Jiangsu, China) were inserted into bilateral Zusanli (ST36) and Kunlun (BL60) acupoints. In rats, the Zusanli (ST36) is located posterior lateral to the knee joint and about 5 mm below the capitulum fibulae. The Kunlun (BL60) is located in the depression between lateral malleolus and achilles tendon of the hind limb [39]. The needles were inserted 5 mm deep into the rats and then stimulated using a HANS electrical stimulation device (Hans-200A, Jisheng Medical Technology, Beijing, China) at 1 mA and 2 Hz. Control and DNP group rats received the same calming procedure without EA stimulation.

#### Statistical analysis

Data are presented as mean ± SEM and were analyzed with SPSS 21.0. All data were analyzed by one-way ANOVA, followed by post hoc test of the least significant difference (LSD) or Dunnett’s post hoc test for multiple comparisons. *P* < 0.05 indicated statistical significance.

## Results

### Generation of a DNP model via large-dose STZ injection

We created a DNP model using a single intraperitoneal STZ injection (65 mg/kg body weight) to trigger type 1 insulin deficiency in rats (Fig. 1a). Relative to controls, diabetic rats exhibited significantly higher FBG and body weight 7 days after diabetes induction (Fig. 1b, c, *P* < 0.01, respectively). Although basal PWTs and PWLs did not differ between groups, they significantly fell in DNP group relative to the control group on day 14 post-STZ injection (D14), which persisted throughout the experiment, indicating development of mechanical allodynia and thermal hyperalgesia (Fig. 1d, f, *P* < 0.01, respectively). Area under the curve (AUC) analysis of PWT and PWL revealed they were significantly lower in DNP rats (Fig. 1e, g, *P <* 0.01 for AUC of PWT, *P* < 0.05 for AUC of PWL). Together, these observations show successful establishment of the type 1 DNP model.

### P2X3 receptor expression is upregulated on L4–6 DRGs in DNP rats

**Fig. 2:**
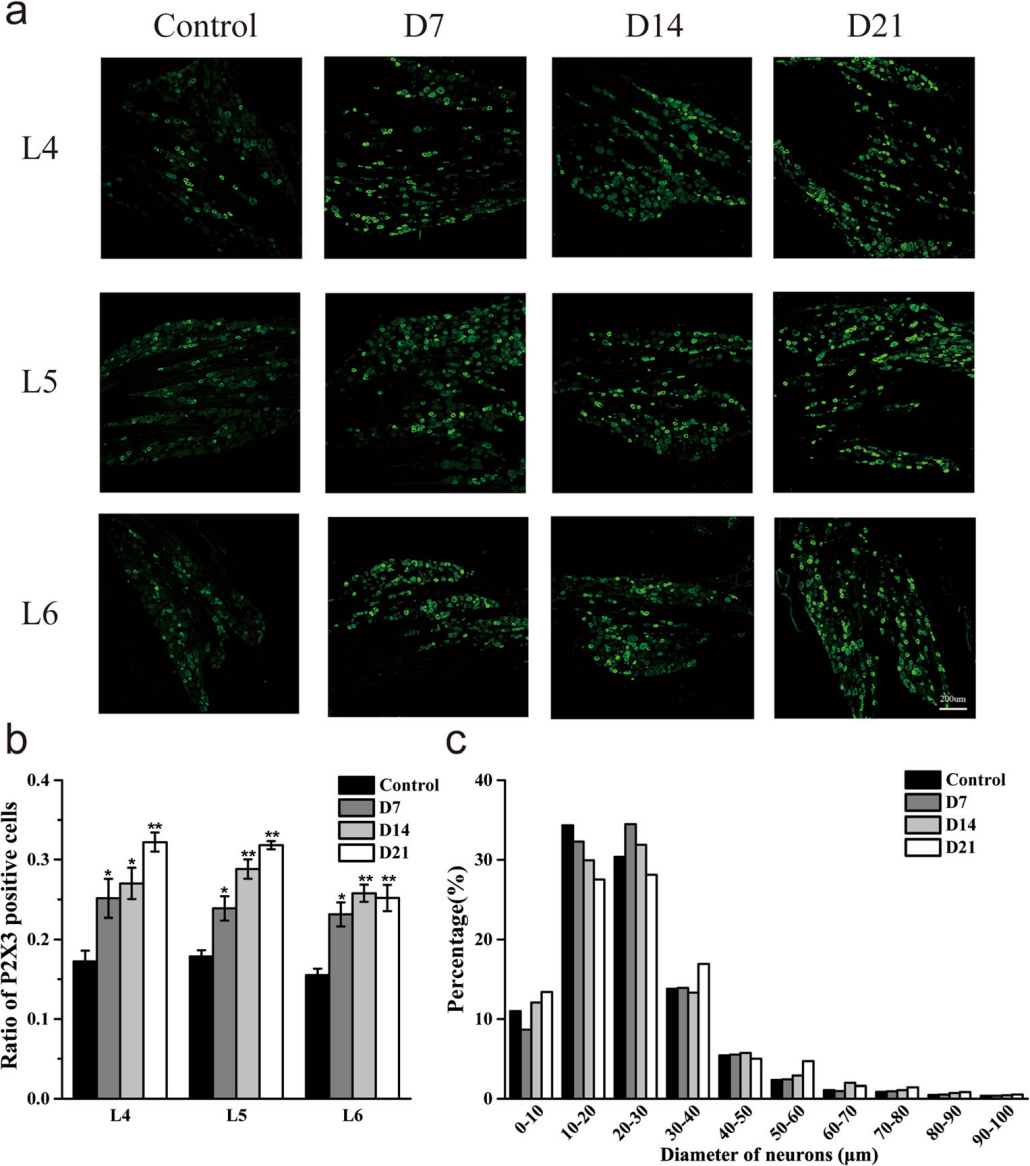
P2X3R expression levels in L4–6 DRG in DNP model. **a** Representative images of L4–6 DRGs from Control, D7, D14, and D21 groups. Scale bars = 200 μm; **b** quantification of P2X3R positive neurons in L4–6 DRGs in different groups; **c **size distribution of P2X3R positive neurons in L4–6 DRGs in different groups. *n* = 3. *P < 0.05, **P < 0.01 vs. control group

We used immunofluorescence to evaluate P2X3R expression in DRG at 7, 14, and 21 days after STZ injection (Fig. 2a). This analysis revealed that relative to control animals, DNP rats had significantly higher P2X3R expression in L4–6 DRG on D7 (*P* < 0.05, respectively), which persisted on D14 (*P* < 0.05 for L4 DRG, *P* < 0.01 for L5 and L6 DRGs) and D21 (*P* < 0.01, respectively) (Fig. 2b). P2X3R was predominantly expressed in DRG neurons with medium and small diameters (< 40 μm) (Fig. 2c).

### P2X3 receptor colocalizes with NeuN, but not with GFAP and CD11 in DRG

**Fig. 3:**
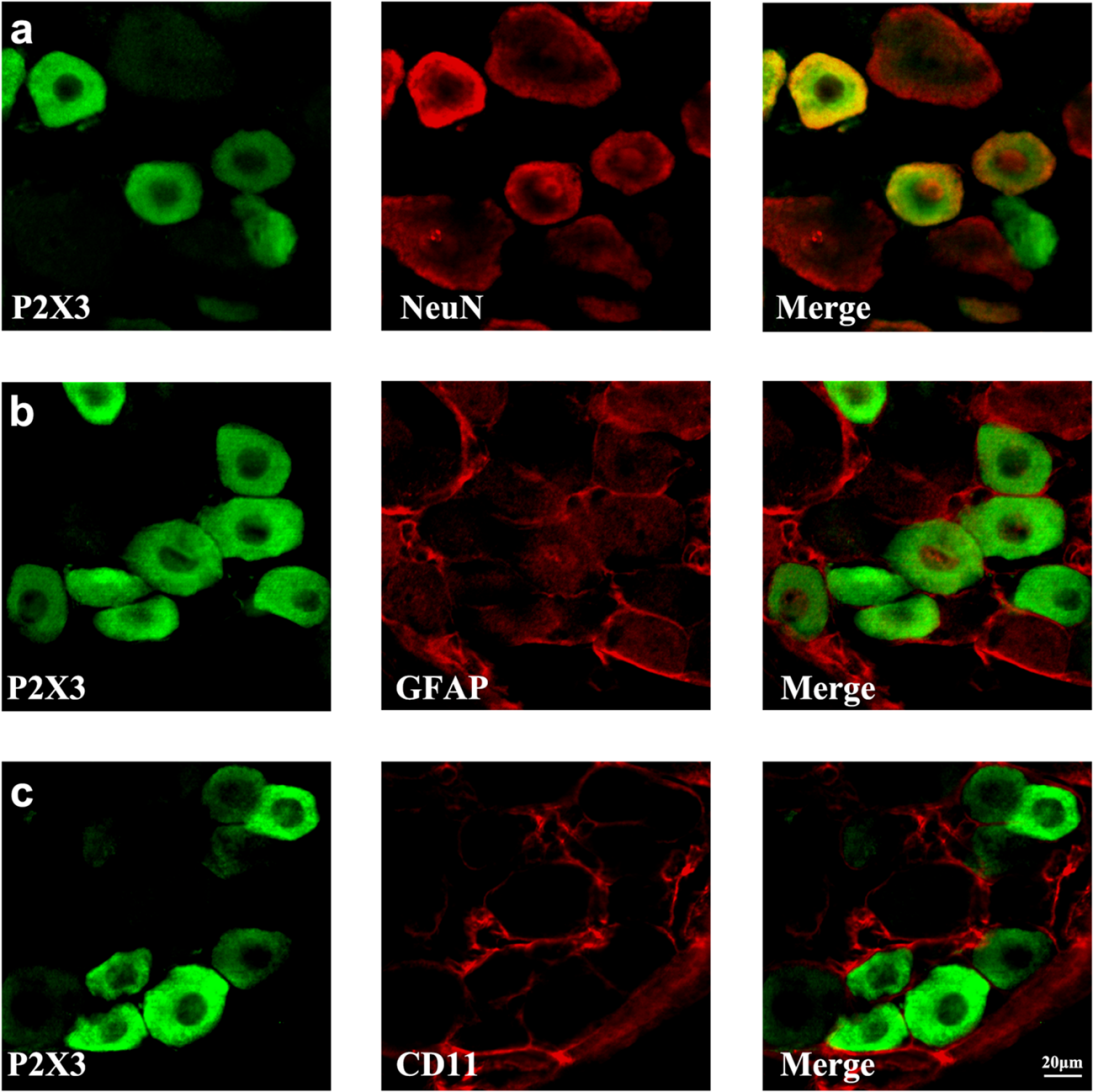
Colocalization immunofluorescence analysis of P2X3 receptor with NeuN, GFAP, and CD11. **a **Immunofluorescence images of P2X3R (green) and NeuN (red) colocalization in DRGs; **b **immunofluorescence images of P2X3 receptor (green) and GFAP (red) colocalization in DRGs; **c **immunofluorescence images of P2X3R (green) and CD11 (red) colocalization in DRGs. Scale bars = 20 μm

We used double immunofluorescence to identify the cell types that express P2X3R in the DRG on D21 and found that P2X3R colocalizes with the neuronal marker NeuN (Fig. 3a), but not with the satellite glial cell marker GFAP (Fig. 3b) or the microglia marker CD11 (Fig. 3c).

### A317491 suppresses diabetic neuropathic pain

**Fig. 4:**
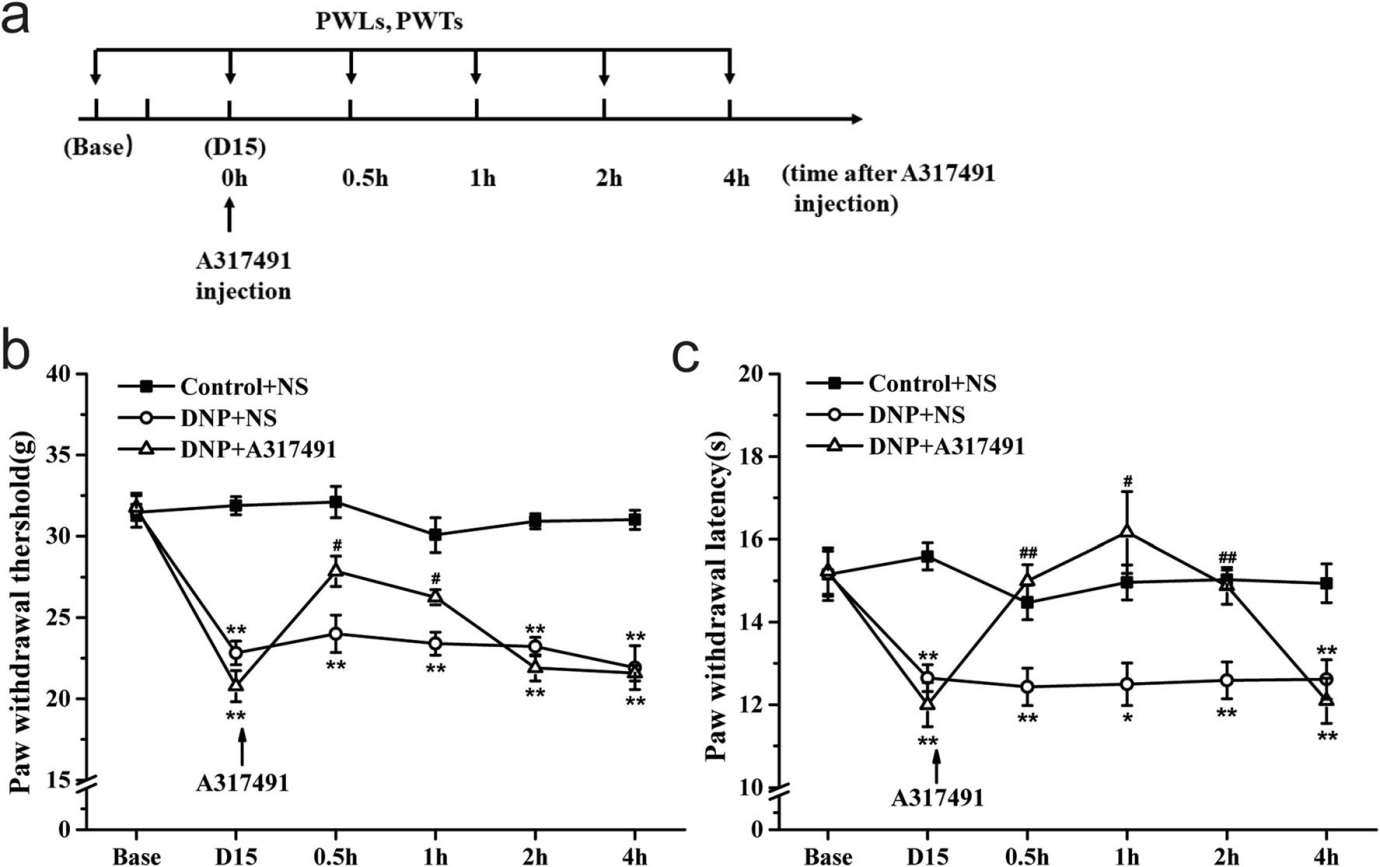
Effects of A317491 on PWT and PWL at different timepoints. **a** Treatment schedule of A317491; **b** effect of A317491 on PWT in DNP rats; **c** effect of A317491 on PWL in DNP rats. Data are shown as means ± SEM, *n* = 6 rats per group. *P < 0.05, **P < 0.01 vs. Control + NS group. ^#^*P* < 0.05, ^##^*P* < 0.01 vs. DNP + NS group

We examined the effect of A317491 on pain behavioral tests in DNP rats vs. controls (Fig. 4a). Prior to A317491 administration, we observed significant differences in PWTs and PWLs in control vs. DNP animals, but not between DNP + NS and DNP + A317491 groups prior to treatment. However, half an hour after A317491 treatment, the DNP + A317491 group showed remarkably milder thermal hyperalgesia and mechanical allodynia relative to the DNP + NS. These effects lasted an hour post A317491 injection for mechanical allodynia and 2 h for thermal hyperalgesia (Fig. 4b, c).

### 2 Hz EA relieves thermal hyperalgesia and mechanical allodynia in DNP rats

**Fig. 5:**
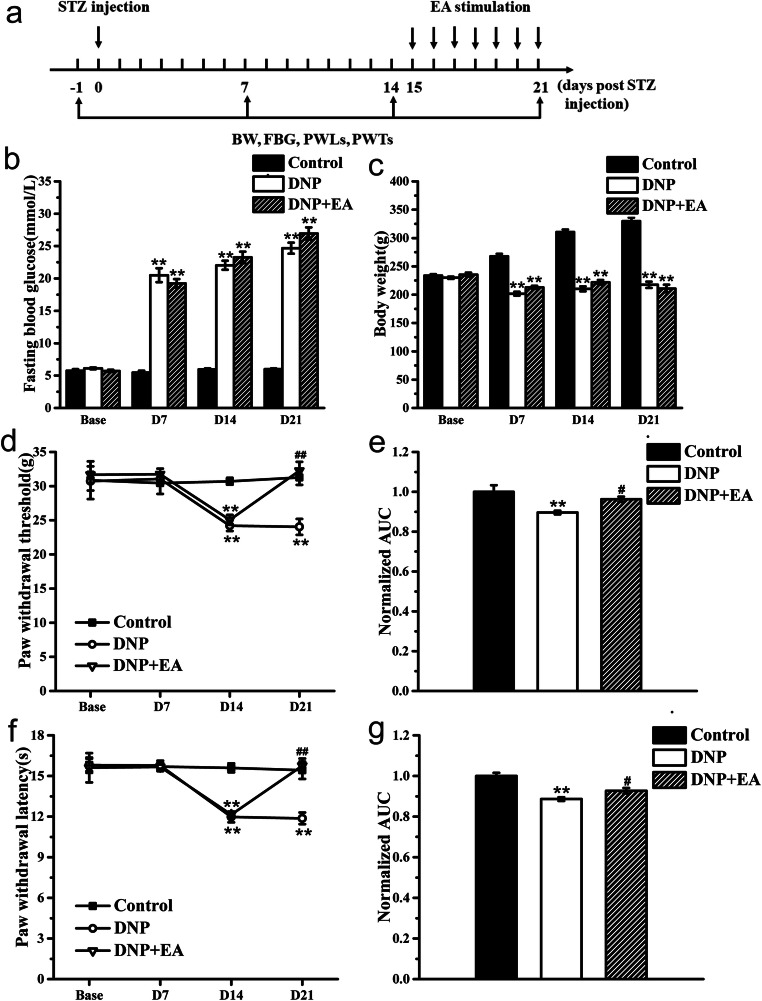
Impact of EA treatment on PWL and PWT in DNP rats. **a** Experimental procedure for generation of the DNP rat model and EA treatment; **b** time course effect of STZ injection and EA treatment on FBG; **c** time course effect of STZ injection and EA treatment on body weight; **d** analgesic effects of 2 Hz EA on PWT; **e** normalized AUC analysis of Fig. 1d; **f** analgesic effect of 2 Hz EA on PWL; **g** normalized AUC analysis of Fig. 1f. *n* = 6 rats per group. **P < 0.01 vs. Control group. ^#^*P* < 0.05, ^##^*P* < 0.01 vs. DNP group

We then established a rat model of STZ-induced DNP and treated all groups with EA following the aforementioned protocols (Fig. 5a). This analysis revealed significantly lower PWT and PWL 14 days after STZ injection (Fig. 5d, f, *P* < 0.01, respectively). Daily 2 Hz EA treatment for 7 days significantly increased PWT and PWL relative to the DNP group at the same time point (Fig. 5d, f, *P* < 0.01, respectively). AUC analysis of PWT and PWL revealed the overall effect of 2 Hz EA on thermal hyperalgesia and mechanical allodynia in DNP rats (Fig. 5e, g, *P* < 0.05, respectively). EA did not affect FBG and body weight relative to the DNP group (Fig. 5b, c).

### 2 Hz EA suppresses P2X3 receptor upregulation in L4–6 DRGs in DNP rats

**Fig. 6:**
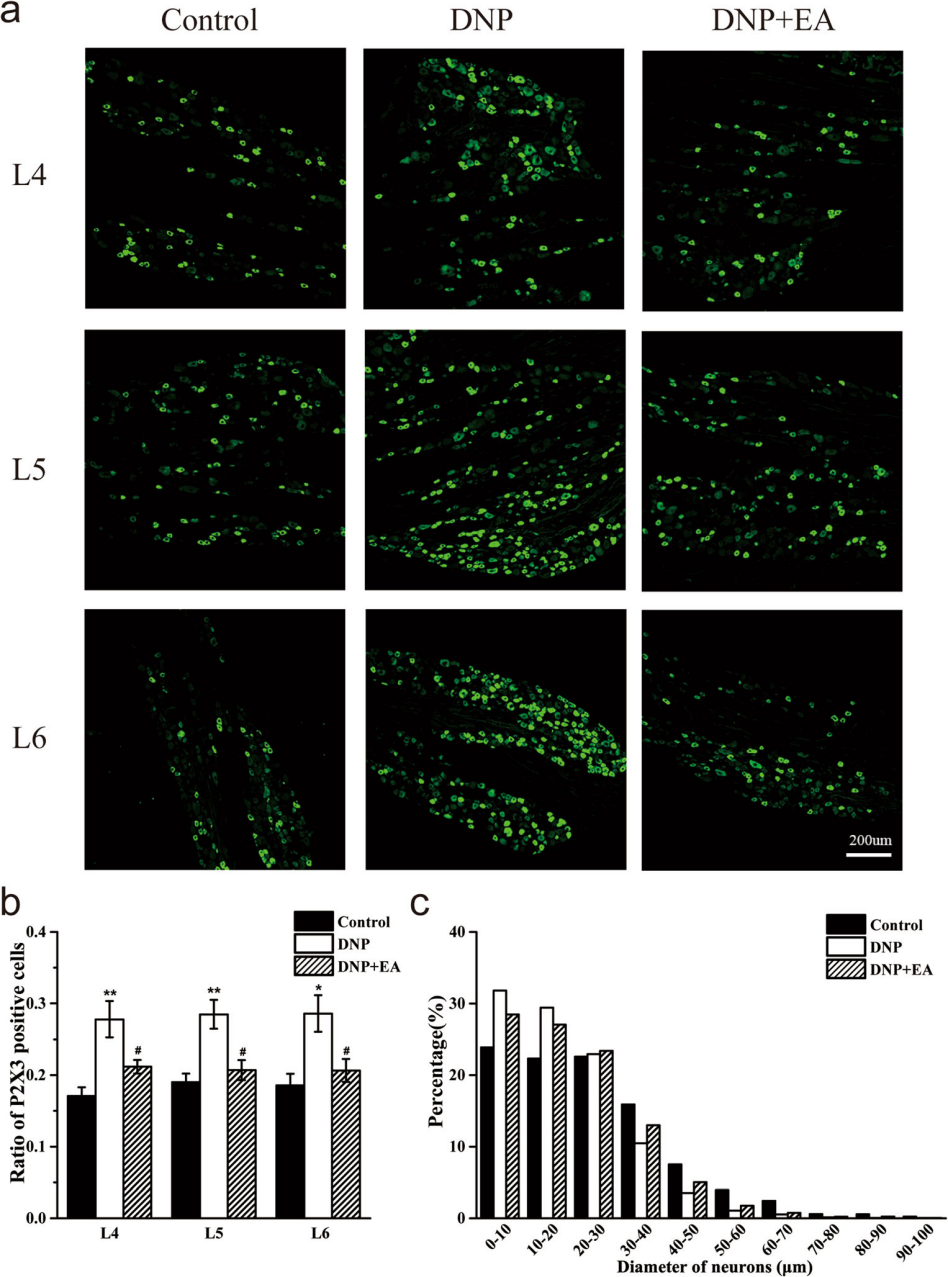
Impact of EA on P2X3R levels in L4–6 DRGs. **a** Representative images of L4–6 DRGs immunofluorescence staining in Control, DNP, and DNP + EA groups. Scale bars = 200 μm; **b** quantification of P2X3R positive neurons in L4–6 DRGs in Control, DNP, and DNP + EA groups; **c** size distribution of P2X3R positive neurons in L4–6 DRGs. *n* = 3. *P < 0.05, **P < 0.01 vs. Control group. ^#^*P* < 0.05 vs. DNP group

To assess whether the analgesic effects of 2 Hz EA on DNP are mediated via P2X3R expression in DRG, we used IF to assess DRG P2X3R expression after continuous 2 Hz EA treatment for 7 days (Fig. 6a). This analysis revealed that P2X3R expression in L4–6 DRGs was markedly increased in the DNP group relative to the control group (Fig. 6b, *P* < 0.01 for L4 and L5 DRGs, *P* < 0.05 for L6 DRG) and was significantly inhibited by 2 Hz EA treatment relative to DNP group (Fig. 6b, *P* < 0.05, respectively). Analysis of P2X3R size distribution in L4–6 DRGs revealed that neither STZ injection nor 2 Hz EA treatment altered the P2X3R distribution (Fig. 6c).

### 2 Hz EA treatment reduces α β-me ATP-induced persistent spontaneous nociception

**Fig. 7:**
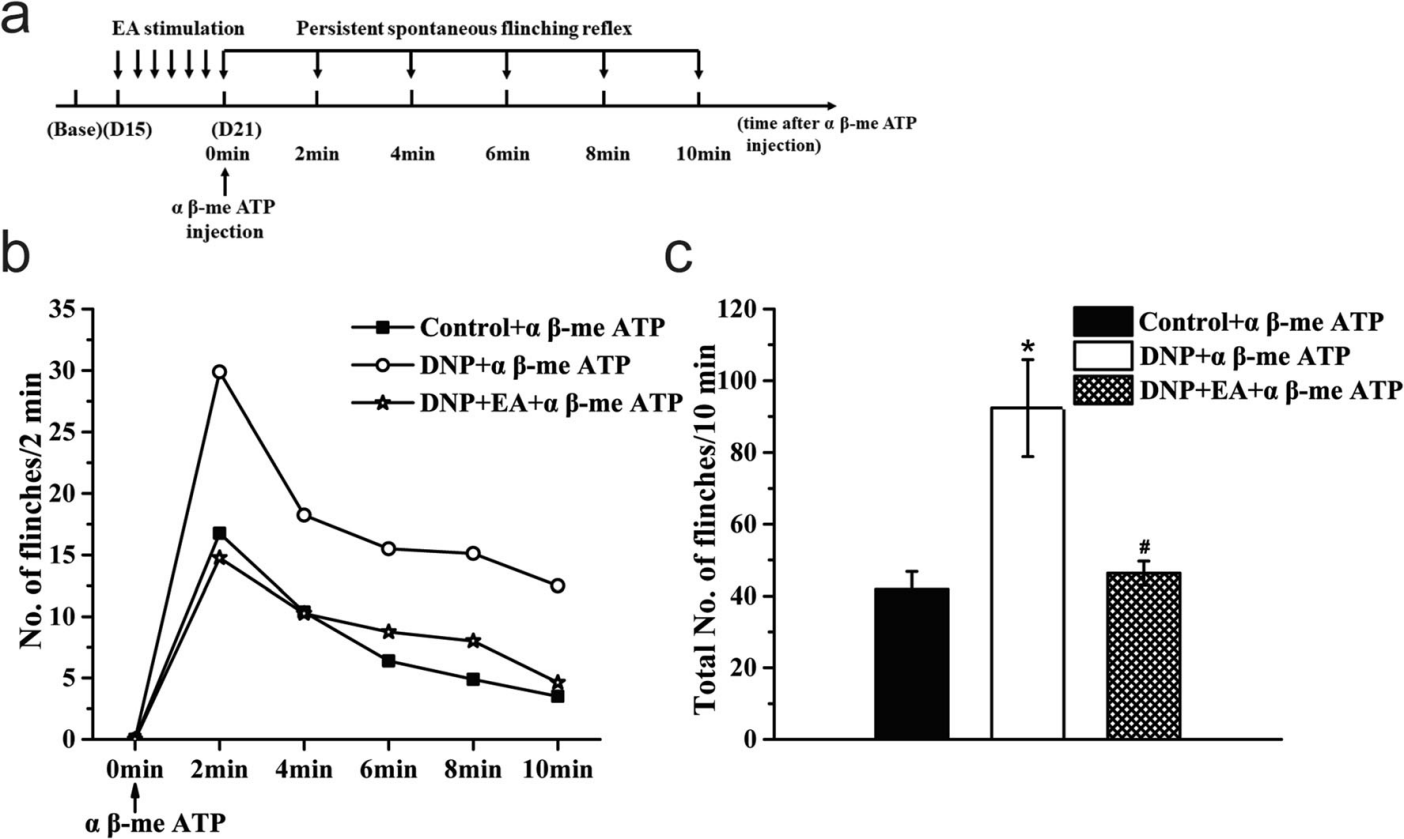
Effects of α β-me ATP on persistent spontaneous flinching reflex. **a** PSN observation schedule after α β-me ATP injection. **b** Number of paw flinches at 2-min time intervals following α β-me ATP injection. **c** Number of paw flinches 10 min after α β-me ATP injection. Data are shown as mean ± SEM, *n* = 8. *P < 0.05 vs. Control + α β-me ATP group; ^#^*P* < 0.05 vs. DNP + α β-me ATP group

Next, we evaluated EA effects on P2X3R-mediated activity. The results described in the preceding sections indicated that the rats developed DNP 14 days after STZ injection. Thus, EA treatment was started on the 15th day and given for 7 consecutive days. On D21, α β-me ATP was injected into the plantar surface of rat left paw immediately after EA treatment to activate P2X3R and elicit persistent spontaneous nociception (PSN) (Fig. 7a). This analysis revealed a reduction in the number of flinches in all groups (Fig. 7b). The number of paw flinches in the EA group significantly increased to 41.88 ± 4.95 after α β-me ATP injection. Animals in the DNP group exhibited more severe PSN after injection, with a marked elevation in the number of paw flinches (92.38 ± 13.52) relative to the control + α β-me ATP group. After EA treatment, DNP rats exhibited α β-me ATP-induced PSN levels similar to those of the control + α β-me ATP group (46.38 ± 3.34 paw flinches) and significantly lower than those of the DNP + α β-me ATP group (Fig. 7c, *P* <0.05).

## Discussion

Here, we investigated the effect of P2X3R expression on diabetic neuropathic pain at various stages of type 1 DNP in a rat model and related mechanisms of EA-mediated analgesic effects. We show that a large dose of STZ injection induces type 1 diabetes in rats. STZ-induced DNP rats exhibited pain hypersensitivities, including mechanical allodynia [40–42], thermal hyperalgesia [43, 44], and spontaneous nociception [45], 14 days after STZ injection, and elevated P2X3R expression in DRGs. We find that 2 Hz EA treatment eliminates pain hypersensitivities and suppresses P2X3R expression. Further analysis found that A317491, a specific P2X3R antagonist, suppressed DNP pain hypersensitivities, while 2 Hz EA analgesic effects were reversed by α β-me ATP, a P2X3 agonist. Taken together, these findings suggest that DNP may be mediated by elevated P2X3R expression and that EA may have analgesic effects via suppression of P2X3R expression in DRGs.

Different approaches have been used to develop DNP animal models. For example, DNP models can be established spontaneously, e.g., the WBN/Kob rat [46] and Ins2 Akita mouse [47], genetically, e.g., the C57BL/Ks (db/db) mouse model [48], through high-fat diets [49], or pharmacologically with alloxan [50] or STZ [51]. Because STZ triggers diabetes features that closely recapitulate those of human diabetes, it is extensively used to generate diabetes models [52]. Some species, including mouse, rat, and monkey, are sensitive to STZ’s cytotoxic effects on pancreatic β cells [53]. STZ may be administrated intraperitoneally (I.P.) or intravenously (I.V.) into adult animals [54]. As higher STZ doses are directly cytotoxic to pancreatic β cells, they are preferred for T1DM induction. These animal models are characterized by reduced islet β cell numbers, non-fasting or fasting hyperglycemia, decreased insulin secretion, and low glucose tolerance during glucose stimulation. Thus, we generated a T1DM rat model using a single, large dose of STZ (65 mg/kg) by i.p. injection. Elevated FBG, gradual weight loss, and pain hypersensitivities indicated successful DNP induction.

Multiple studies have shown P2X3 receptor activity involvement in DNP [55, 56]. Diabetes-induced damage to peripheral tissues [57] may sensitize sensory neurons or nociceptors through various mechanisms. Elevated neurotransmitter release and peptide co-release with ATP are thought to cause peripheral and central sensitization [51, 58]. Because P2X3R is sensitive to nanomolar ATP concentrations, purinergic signal transduction can be adjusted using varying ATP concentrations [59]. ATP modulates pain sensitivity via changes in sensory neuron voltage-gated ion channels activity, including CaV and NaV [60, 61]. The P2X3R agonist, α β-me ATP triggers fast desensitizing inward current, while TNP-ATP significantly blocks inward currents in rat DRGs [62]. Pain hypersensitivity in STZ-induced diabetic rats can also be reduced by treatment with A317491, a P2X3R antagonist, and TNP-ATP [12, 13, 63], indicating that peripheral P2X3 receptors mediate neuropathic pain.

Although the analgesic effects of EA are well documented, its mechanisms are poorly understood. Present studies have indicated that EA may improve insulin resistance [64, 65] by enhancing insulin sensitivity in rats [66]. Previously, we have shown that both 2 Hz and 100 Hz EA stimulation relieve DNP, but 2 Hz EA has a stronger analgesic effect [67]. Acupuncture triggers ATP release by keratinocytes and subcutaneous mast cells [68–70]. This contributes to transient cytoskeleton reorganization and elevates intracellular Ca^2+^ concentration, stimulating other signaling pathways [71]. EA and gentle manual rotation of acupuncture needles have been reported to alter extracellular ATP concentration. However, these ATP levels are not sufficiently high to activate P2X3R due to rapid ATP degradation, explaining the absence of direct pain during EA treatment [72]. α β-me ATP is reported to elicit nociceptive responses [73] and block EA analgesic effects [30], which is consistent with our findings. We find that neuropathic pain in DM rats rises with increased P2X3R expression and that EA suppresses P2X3R expression on DRGs. Indicating peripheral P2X3R involvement in EA-mediated pain attenuation. However, mechanisms by which ATP upregulates P2X3R expression in DNP are unclear.

## Conclusions

In summary, we have shown the relationship between pain sensitization and P2X3R expression in DRG of DNP rats. We find that EA mediates analgesia by downregulating P2X3R expression. This study provides rationale for clinical DNP treatment using EA.

## Abbreviations

DNP: Diabetic neuropathic pain
P2X3R: P2X3 receptor
DRG: Dorsal root ganglion
ATP: Adenosine 5′-triphosphate
STZ: Streptozotocin
FBG: Fasting blood glucose
BW: Bodyweight
EA: Electroacupuncture
PWL: Paw withdrawal latency
PWT: Paw withdrawal threshold
PSN: Persistent spontaneous nociception
